# Identification of genetic risk variants for Type-2 Diabetes mellitus in Pakistani Pashtun population: A case-control association study

**DOI:** 10.12669/pjms.40.10.10292

**Published:** 2024-11

**Authors:** Asif Jan, Ramzi A. Mothana, Jun-Ya Kaimori, Tahir Muhammad, Mehtab Khan, Syed Shaukat Ali, Naveed Rahman, Abdullah R. Alanzi

**Affiliations:** 1 Asif Jan, District Headquarter Hospital (DHQH) Charsadda, Charsadda 24430, Pakistan. Department of Pharmacy, University of Peshawar, Peshawar 25000, Pakistan; 2 Ramzi A. Mothana, Department of Pharmacognosy, College of Pharmacy, King Saud University, Riyadh 1151, Saudi Arabia; 3 Jun-Ya Kaimori, Department of Nephrology, Osaka University Graduate School of Medicine, Suita, 2-2 Yamadaoka, Suita, Osaka 565-0871, Japan. Institute of Medical Science, University of Toronto, Toronto 43964, ON, Canada; 4 Tahir Muhammad, Molecular Neuropsychiatry & Development (MiND) Lab, Campbell Family Mental Health, Research Institute, Centre for Addiction & Mental Health, Toronto 43964, ON, Canada; 5 Mehtab Khan, Department of Biology, Faculty of Science, University of Moncton, Canada; 6 Syed Shaukat Ali, Department of Pharmacy, University of Malakand, Pakistan; 7 Naveed Rahman, Department of Pharmacy, University of Peshawar, Peshawar 25000, Pakistan; 8 Abdullah R. Alanzi, Department of Pharmacognosy, College of Pharmacy, King Saud University, Riyadh 1151, Saudi Arabia

**Keywords:** Pashtun, Pakistan, Type-2 Diabetes Mellitus (T2DM), Exome Sequencing (ES), Genotyping, MassARRAY, Risk variants

## Abstract

**Background and Objective:**

Pakistan, a South Asian developing country, is experiencing a rapid increase in number of diabetes cases. High prevalence ratio of diabetes in Pakistani population and lack of genetic research studies prompted us to design this study. This present study investigated Pakistani Pashtun population for (known and novel SNPs) and its possible correlation with Type-2 Diabetes Mellitus (T2DM).

**Methods:**

This two stage (discovery & validation stage), case-control association study included one thousand individuals (Patients with T2DM=500 & controls=500) from eight districts of Khyber Pakhtunkhwa Pakistan. The study duration/period was from March 2018 to January 2020. In the first stage (the discovery stage) the target population was screened for known and novel T2DM-associated genetic markers. In the validation stage, identified variants were confirmed for T2DM association using MassARRAY genotyping and association analysis.

**Results:**

Exome sequencing detected eleven known and four novel/new genetic markers in the study population. Novel variants were preferred over the known for follow-up analysis/validation. Among the identified variants strong associations were confirmed for the following variants; rs1781133/*ANKRD65* (OR=2.10, 95%Cl=1.06–3.08, P=0.003) rs2274791/*TTLL10* (OR=1.97, 95%Cl=1.36-2.62, P=0.025), rs71628928/*RNF223* (OR=1.82, 95%Cl=0.97-1.92, P=0.041), and rs609805/*SCNN1D* (OR=2.21, 95%Cl=1.92-3.09, P=0.001) with T2DM; other reported variants showed no noticeable association (having P>0.05) with T2DM.

**Conclusion:**

This study reports new genetic risk variants for T2DM in Pashtun population providing valuable insights into the genetic basis of T2DM in this group.

## INTRODUCTION

Pakistan a developing South Asian country is facing a sharp increase in number of diabetic cases. In the year 2019 roughly 19.4 million peoples in Pakistan were having diabetes and the cases are expected to increase to 34.4 million by 2030 and 37.1 million by 2045. Key contributors to high number of diabetes cases includes genetic and environmental factors. Variations in genes occur due to environmental changes and alteration and or mutation in human genes that further affect disease occurrence/disease susceptibility.^1^,^2^

In the past greater number of studies have been conducted in various cohort, to unmask the complex genetic architecture of T2DM. New OMIC technologies and other advanced technologies have discovered greater than 400 loci responsible for T2DM.^3^ However large proportion of these studies disproportionately carried out in individuals of European origin and very few in Pakistani cohorts.^4^ It is believed that the genetic spectrum of diabetes in Pashtun population of Pakistan is different from elsewhere. The unique genetic makeup and unique living style of the study cohort make it very suitable for genetic studies as of present.^5^ The present study was initiated to screen Pashtun ethnic population of Pakistan, for known (previously associated with T2DM) and novel genetic biomarkers using Whole Exome Sequencing (WES).

## METHODS

**Fig.1:**
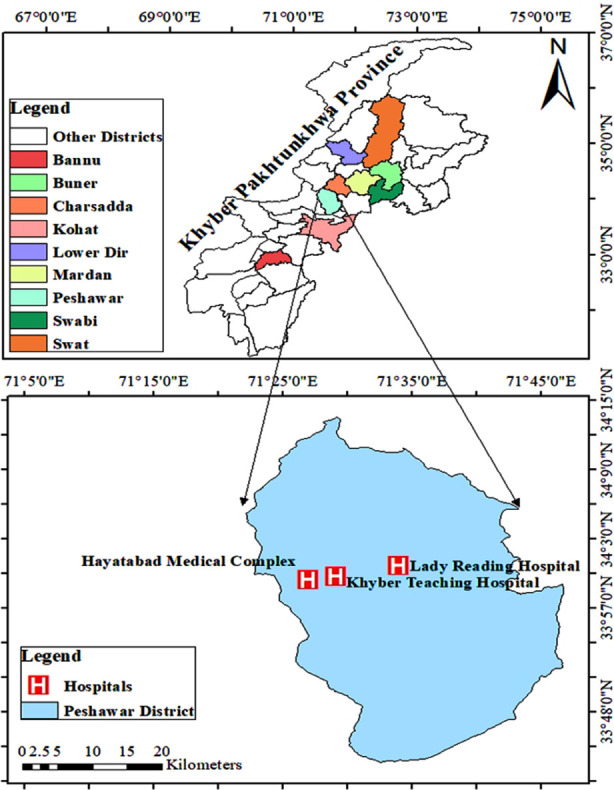
Map representing different districts from where the study participants belongs and major hospital where blood samples were collected.

In this case control association study, a total of n=1000 individuals (500 diabetic patients & 500 healthy volunteers) from Pakistani Pashtun population were enrolled. The study duration was from March 2018 to January 2020.The participants were from eight districts of KP (Khyber Pakhtunkhwa) Pakistan. It was carried at multiple health centers/hospitals; patients were enrolled at Hayatabad Medical Complex Peshawar, Khyber Teaching Hospital Peshawar and Lady Reading Hospital Peshawar (Fig.1). Blood samples from healthy volunteers/controls were collected at free medical screening camps arranged by Non-Government Organization (NGO) at Hayatabad Medical Complex Peshawar and Charsadda Diabetes Association. Informed consent from all participants was obtained, ensuring their willingness to participate in the study. Detailed information (demographic and clinical data) of both cases and control were obtained on a proforma.

### Ethical Approval:

The study was approved by the ethical committee of the Department of Pharmacy at the University of Peshawar, under approval number 907/PHAR, Date: October 30, 2018. Consent to participate in the study was taken from all the study subjects.

### Inclusion Criteria:

- Diabetes mellitus diagnosed as per indications settled by International Diabetes Federation (IDF); i.e. fasting blood glucose level (shortly denoted as FBS) >126 mg/dL and RBS (random blood glucose level) >200 mg/dL.
- Individuals with age in range of 30 to 80 years.
- Study participants from Pashtun cohort.

### Exclusion Criteria:

- Study Participants with chronic illnesses such as recent severe infections like hepatitis or coronavirus or malignancies were not enrolled in the study.
- Mentally ill patients.
- Those patients having age not in range (30-80 years).

The control group (without having any major disease/pathology) comprised healthy volunteers from the general population and were matched in terms of age and gender with patients. The FBS and RBS levels of the control group were in normal range, i.e. FBS less than 100 mg/dL and RBS less than 200 mg/dL.

### Blood Samples Collection:

A blood sample of 3ml was collected from each study individual and drawn into EDTA tubes. Proper labelling of the tubes was ensured to maintain accurate identification of the samples. Following this EDTA containing blood samples were stored at -10°C.

### DNA Extraction and Quantification:

Two hundred micro-liters (200 μl) of whole blood samples were used for DNA extraction. Extraction was carried out using kit method (specification of the kit used: Wiz-Prep no. W54100) whereas DNA quantification was carried out using the Invitrogen Qubit™^3^ system. Finally, DNA concentration was adjusted to 5 ng/μL.

### DNA Pooling:

DNA samples pooling was carried using carefully following the previously described protocols.^6^,^7^ Two pools were constructed; the first pool consisted of DNA samples from 500 individuals diagnosed with T2DM, while the second pool contained DNA samples from 500 healthy controls. Pools were constructed in such a way that it contains equal amount of DNA (10ng) from every participant. Pooling helped us in reducing the number of individual DNA samples that need to be processed. The DNA pools constructed were then subjected to high-throughput sequencing, for identification of genetic variants.

### Whole Exome Sequencing:

Exome Sequencing was performed at center of genomics, Rehman Medical Institute Peshawar. The aim of exome sequencing was to capture variants in protein-coding region (the exome) which is believed to contain a significant portion of disease-related genetic variants. Using the Illumina Nextera XT DNA library preparation kit paired-end libraries of the pooled DNA samples were generated. This kit provides a streamlined and efficient workflow for library preparation, enabling the capture of the exonic regions of interest. The quantified DNA libraries, representing the pooled samples, were then subjected to sequencing using the Illumina HiSeq2500 sequencing machine.

### Bioinformatic Analysis:

To process the raw sequencing data a custom-built, in-house next-generation sequencing bioinformatics pipeline was used. The analysis workflow involved several steps to ensure data quality and accurate variant identification. First, the FAST-Q files generated by HiSeq2500 sequencing platform were subjected to a quality control step. Low-quality reads, defined as those with a quality score (Q-score) below 30, were filtered out using the Trimmomatic software tool.^8^ Next, RAMICS software was used to align the filtered reads to reference genome (hg19/GRCh37) assembly.^9^ This alignment process allowed for mapping the reads to their corresponding genomic locations. For variant calling was GATK and SAM software tools were used.^10^,^11^ These tools employ different algorithms and strategies to identify genetic variants, such as SNPs and small insertions/deletions (indels), in the aligned reads. To annotate the identified variants, the ANNOVAR software tool was employed.^12^ The ANNOVAR generated annotated variant list, was stored as a Comma-Separated Values (CSV) file format. For easy interpretation and visualization of the data, the CSV file was copied into an Excel format sheet. Excel provides a user-friendly interface for filtering, viewing, searching and interpreting the annotated variant data, allowing us to easily explore the genetic variants of interest.

### Variants filtration and validation by genotyping:

Whole Exome Sequencing (WES) generated large amount of data (>600 GB of data), that poses challenges in terms of handling and processing. To efficiently manage and focus on the variants of interest, a filtering strategy was implemented. Initially, the annotated file containing variant information was manually curated. Novel, exonic non-synonymous missense variants were retained for further analysis. These types of variants have the potential to cause functional changes in proteins or affect the regulation of gene expression, making them relevant to the study. On the other hand, synonymous variants, which do not alter the amino acid sequence of proteins, were excluded from the analysis.

Synonymous variants are generally considered to have a neutral or benign impact on protein function and may not directly contribute to the phenotype under investigation. By filtering the data in this manner, the focus was narrowed down to variants that are more likely to be functionally relevant and potentially associated with T2DM. Following the filtration step; validation of WES identified variants were carried out using Sequenom MassARRAY genotyping and association analysis. The MassARRAY system is a robust and high-throughput genotyping platform known for its accuracy and efficiency. It enables the genotyping of multiple single nucleotide polymorphisms (SNPs) simultaneously, ranging from tens to hundreds of SNPs, within a short period of time.

### Statistical Analysis:

For data analysis latest statistical software the IBM SPSS.V24 (version 24) was used. The important variables selected for analysis, includes gender, age of the subjects, geographical area, weight, exercise habits, lifestyle factors, diet and variants identified/reported. To assess the differences in risk allele frequencies distribution between cases & controls, the χ^2^ test was employed. Whereas T2DM×Variants association was evaluated applying binary logistic regression analysis. A probability value less than 0.05 was considered statistically significant.

## RESULTS

The socio-demographics of the study participants have been described in Table-I. Co-morbidities prevalence was recorded and cases compared with controls. Table-II. The mentioned co-morbidities include hypertension (high blood pressure), renal failure (kidney failure), hypercholesterolemia (high cholesterol levels), and retinopathy (eye disease). Majority of the patients included in the study were reported as physically inactive, indicating that they were not involved in regular physical activity. Moreover, the study participants predominantly resided in urban areas of Khyber Pakhtunkhwa Pakistan. The study participant’s compliance with prescribed medications and dietary recommendations was recorded as poor. Majority of the participants did not adhere well to their prescribed drug regimens and dietary guidelines.

**Table-I:** Socio-demographic details and other characteristics of study Participants.

Variables	Case n(f)	Control n(f)	P-value
***Gender***			0.897
Male	358 (71.6%)	356 (71.2%)	
Female	142 (28.4%)	144(28.8%)	
Mean age (Years)	57±12.40	57±13.43	0.951
Mean weight (kg)	61.64±6.07	59.55±8.32	0.801
***Occupation***			0.589
Labour	140 (28.0%)	119 (23.8%)	
Government servant	30 (6.0%)	40 (8.00%)	
Business man	10 (2.00%)	08 (1.60%)	
Farmer	20 (2.00%)	90 (18.0%)	
House wife	130 (26.0%)	142 (28.4%)	
Driver	80 (16.0%)	40(8.00%)	
Shopkeeper	90 (18.0%)	61(12.2%)	
***Geographical area (District)***			0.439
Peshawar	150 (30.0%)	139 (27.8%)	
Charsadda	70(14.00%)	82 (16.4%)	
Swat	19(3.80%)	11 (2.20%)	
Dir	11 (2.20%)	09 (1.80%)	
Mardan	120(24.0%)	150 (30.0%)	
Kohat	15 (3.00%)	08 (1.60%)	
Bannu	25 (5.00%)	19 (3.80%)	
Buner/Behlola	90(18.00%)	82(16.40%)	
***Family history of T2DM***			0.02
Yes	475 (95.0%)	55 (11.00%)	
No	25 (5.00%)	445(89.00%)	
***Exercise***			0.11
Non-exercising	411 (82.2%)	370 (74.0%)	
Walking	75 (15.0%)	98 (19.06%)	
Jogging	09 (1.80%)	22(4.40%)	
Gym/Sport	05 (1.00%)	10 (2.00%)	
***Smoking***			0.62
Cigarette	113 (22.6%)	98 (19.06%)	
Snuff	220 (44.0%)	240 (48.0%)	
No-smoking	167 (33.4%)	162 (32.4%)	
***Diet control/compliance***			0.43
Yes	290 (58.0%)	311 (62.2%)	
No	210 (42.0%)	189 (37.8%)	

N= number; F=frequency; Kg=kilogram.

**Table-II:** Co-morbidities prevalence in patients and healthy volunteers.

Disease	Frequency		P-value

	Cases	Control	
Hypertension	64.00%	10.00%	0.002
Retinopathy	61.00%	0.00%	< 0.001
Hypercholesterolemia	14.00%	6.01%	0.009
IHD	24.00%	0.00%	< 0.001
Renal failure	12.00%	0.00%	< 0.001
HCV	0.00%	0.00%	< 0.001
HBV	0.00%	0.00%	< 0.001

***Abbreviations:*** HCV: Hepatitis C virus; HBV: Hepatitis B virus; IHD: Ischemic heart disease

### WES Results:

The exome sequencing reported a total of: n = 1,248,875 Single Nucleotide Polymorphisms (SNPs). Among the total SNPs, n = 691,223 were reported as heterozygous, in a heterozygous SNPs, one allele differs from the other at particular location. There were n = 407,572 homozygous SNPs, in homozygous SNPs both alleles at the specific genomic position were the same. A total of n = 74,390 insertions were identified, indicating the addition of extra nucleotides at specific positions in the genome. The exome sequencing identified n = 99,392 deletions, which indicates the removal of nucleotides from specific positions in the genome. Out of the total SNPs, n = 50,280 were located in exonic SNPs, exon is protein coding regions. Within the exonic SNPs, n = 7,910 were reported as missense variants. Missense variants are SNPs that result in the replacement of single amino acid in the encoded protein. Among the exonic SNPs, n = 1,987 variants were expressed in the pancreas. This suggests that these SNPs are present in the genomic regions that are actively transcribed and translated in pancreatic tissues. A total of n = 650 SNPs were reported as pathogenic. Pathogenic variants are genetic variations that are associated with the development or increased risk of a disease or disorder.

Exome sequencing in the target population identified a long list of genes and polymorphism present in it. WES identified; known genetic markers in *NOTCH2, GCKR, NEUROD1, CAPN10, ADCY5, WFS1, CDKAL1, HLA-B, GCK, PAX4* and *SLC30A8* genes (Table-III). Genetic variants/markers in these genes have been previously reported in literature for T2DM association. The novel mutations/variants reported in the study population were rs904589/*ANKRD65*, rs1781133/*ANKRD65*, rs145378993/*ANKRD65*, rs13374146/*TTLL10*, rs1320571/*TTLL10*, rs2274791/*TTLL10*, rs71628928/*RNF223*, rs4333796/*RNF223*, rs609805/*SCNN1D*, rs6690013/*SCNN1D* and rs2228579/*SCNN1D*. These variants have not previously been reported for any disease by any research study or database. Of these novel variants WES labeled/marked rs1781133/*ANKRD65*, rs2274791/*TTLL10*, rs71628928/*RNF223*, and rs609805/*SCNN1D* as daibetogenic/pathogenic or disease causing (Table-IV).

**Table-III:** Whole exome sequencing identified variants in already known genes for T2DM.

Gene	dbSNP ID	Variant	Chr	Genotype	RAF cases	RAF controls	Consequence	Sift Predication	PolyPhen Predication	HGVSc	HGVSp
NOTCH2	rs61788900	T>T/C	1	het	97.21	87.91	missense_variant	Tolerated	Benign	c.137A>G	p.Asn46Ser
NOTCH2	rs61788901	C>C/T	1	het	21.43	23.06	missense_variant	Tolerated	Benign	c.112G>A	p.Glu38Lys
NOTCH2	rs11810554	G>G/C	1	het	71.54	69.11	missense_variant	Tolerated	Benign	c.57C>G	p.Cys19Trp
GCKR	rs1260326	T>T/C	2	het	55.32	50.93	missense_variant	Tolerated	Benign	c.1337T>C	p.Leu446Pro
NEUROD1	rs1801262	T>C/C	2	hom	45.16	28.21	missense_variant	Deleterious	Possibly Damaging	c.133A>G	p.Thr45Ala
CAPN10	rs7607759	A>A/G	2	het	65.68	30.90	missense_variant	Deleterious	Damaging	c.1510A>G	p.Thr504Ala
CAPN10	rs55878652	T>T/C	2	het	76.89	35.22	splice region variant	Deleterious	Damaging	c.1990-7T>C	------
CAPN10	rs2975766	A>G/G	2	hom	79.93	47.13	missense_variant	Deleterious	Damaging	c.1996A>G	p.Ile666Val
ADCY5	rs4482616	A>A/G	3	het	94.78	52.91	splice region variant	Deleterious	Damaging	c.2900+4T>C	---
WFS1	rs1801212	G>G/A	4	het	61.39	49.14	missense_variant	Deleterious	Damaging	c.997G>A	p.Val333Ile
WFS1	rs1801208	G>G/A	4	het	52.63	31.76	missense_variant	Deleterious	Damaging	c.1367G>A	p.Arg456His
CDKAL1	rs77152992	C>C/T	6	het	47.21	32.11	missense_variant	Deleterious	Damaging	c.1226C>T	p.Pro409Leu
HLA-B	rs2308655	C>C/G	6	het	61.43	31.06	missense_variant	Tolerated	Probably Damaging	c.1046G>C	p.Cys349Ser
HLA-B	rs1051488	C>C/T	6	het	51.54	45.12	missense_variant	Tolerated	Benign	c.985C>T	p.Ala329Thr
GCK	rs2908274	G>G/A	7	het	35.32	32.93	upstream_gene_variant	Tolerated	Benign	---	----
PAX4	rs712701	T>T/G	7	het	45.16	38.01	missense_variant	Deleterious	Possibly Damaging	c.962A>C	p.His321Pro
PAX4	rs772936097	TA>TA/T	7	het	65.68	34.44	splice region variant	Deleterious	Possibly Damaging	c.748-3delT	----
SLC30A8	rs13266634	C>C/T	8	het	76.89	68.21	missense_variant	Tolerated	Benign	c.973C>T	p.Arg325Trp

***Abbreviations:*** SNP: Single nucleotide polymorphism; Chr: Chromosomes; Freq: frequency; HGVSc: the HGVS coding sequence name; HGVSp: the HGVS protein sequence. Het: Heterozygous; Homo: homozygous; RAF: risk allele frequency.

**Table-IV:** Whole Exome Sequencing identified new/novel variants in the target population.

Gene	dbSNP ID	Variant	Chr	Genotype	Risk allele Freq (cases)	Risk allele Freq (controls)	Consequence	Sift	PolyPhen	HGVSc	HGVSp
ANKRD65	rs904589	C>C/G	1	het	37.21	32.98	missense_variant	Tolerated -	Unknown	c.1165G>C	p.Glu389Gln
ANKRD65	rs1781133	C>C/A	1	het	41.43	21.76	missense_variant	Deleterious	Damaging	c.599G>T	p.Gly200Val
ANKRD65	rs145378993	C>C/T	1	het	21.54	20.12	missense_variant	Tolerated	Benign	c.386G>A	p.Arg129His
TTLL10	rs13374146	T>T/C	1	het	25.32	22.93	missense_variant	Tolerated	Benign	c.746T>C	p.Val249Ala
TTLL10	rs1320571	G>G/A	1	het	35.16	28.21	missense_variant	Tolerated	Benign	c.1343G>A	p.Ser448Asn
TTLL10	rs2274791	G>G/A	1	het	45.68	30.44	missense_variant	Deleterious	Damaging	c.1733G>A	p.Gly578Asp
RNF223	rs71628928	G>G/T	1	het	66.89	45.22	missense_variant	Tolerated	Possibly Damaging	c.725C>A	p.Pro242His
RNF223	rs4333796	G>G/A	1	het	69.93	67.12	missense_variant	Tolerated	Benign	c.515C>T	p.Ala172Val
SCNN1D	rs2228579	G>G/C	1	het	24.78	22.90	missense_variant	Tolerated	Benign	c.1630G>C	p.Glu544Gln
SCNN1D	rs6690013	G>G/A	1	het	41.39	39.54	missense_variant	Tolerated	Benign	c.2176G>A	p.Gly726Ser
SCNN1D	rs609805	G>G/A	1	het	42.63	21.76	missense_variant	Deleterious	Damaging	c.2308G>A	p.Gly770Arg

***Abbreviations:*** SNP: Single nucleotide polymorphism; Chr: Chromosomes; Freq: frequency; HGVSc: the HGVS coding sequence name; HGVSp: the HGVS protein sequence. Het: Heterozygous; Homo: homozygous.

### SNPs validation and association analysis:

To validate WES results and reduce the chances of false-negative and false-positive identification rates, the prioritized (novel) SNPs identified by exome sequencing were genotyped using MassARRAY technology. MassARRAY is a genotyping platform that allows for accurate and high-throughput genotyping of specific genetic variants. After genotyping the SNPs, association analysis using appropriate statistical tests was performed. Using association analysis of the genotyped SNPs, we assessed the associations between the SNPs and T2DM susceptibility. Genotyping and follow-up association analysis showed strong association of rs1781133/*ANKRD65* (OR =2.10, 95%Cl = 1.06–3.08, P = 0.003), rs2274791/*TTLL10* (OR = 1.97, 95%Cl = 1.36-2.62, P = 0.025), rs71628928/*RNF223* (OR = 1.82, 95%Cl = 0.97-1.92, P = 0.041), and rs609805/*SCNN1D* (OR = 2.21, 95%Cl = 1.92-3.09, P = 0.001) with T2DM in the study population (Table-V). These SNPs exhibited significant differences in the distribution of Risk allele frequencies (RAFs) between cases and healthy volunteers (controls). The higher burden of risk alleles in patients compared controls suggests a potential contribution of these genetic variants to the development or increased risk of T2DM in the studied population. Likewise, variants rs904589/*ANKRD65*, rs145378993/*ANKRD65*, rs13374146/TTLL10, rs1320571/*TTLL10*, rs4333796/RNF223, rs6690013/*SCNN1D* and rs2228579/*SCNN1D* when checked for T2DM showed no/negative (P > 0.05) association with T2DM in the target population.

**Table-V:** Conformation of WES identified Prioritized/novel variants in the 2^nd^ (validation) stage.

Gene	SNP	Alteration	RAF (cases )	RAF (controls)	OR (95% CI)	P-value
ANKRD65	rs904589	C>C/G	0.33	0.29	1.74 (1.40–1.81)	0.090
ANKRD65	rs1781133	C>C/A	0.41	0.21	2.10 (1.06–3.08)	0.003
ANKRD65	rs145378993	C>C/T	0.23	0.20	1.27 (0.87–1.67)	0.134
TTLL10	rs13374146	T>T/C	0.35	0.31	1.62 (1.08-1.76)	0.126
TTLL10	rs1320571	G>G/A	0.33	0.28	1.53 (0.56–1.95)	0.081
TTLL10	rs2274791	G>G/A	0.45	0.30	1.97 (1.36-2.62)	0.025
RNF223	rs71628928	G>G/T	0.66	0.55	1.82 (0.97-1.92)	0.041
RNF223	rs4333796	G>G/A	0.69	0.67	1.15 (0.87–2.13)	0.291
SCNN1D	rs2228579	G>G/C	0.24	0.22	1.77 (0.83-1.82)	0.215
SCNN1D	rs6690013	G>G/A	0.41	0.39	1.46 (1.32-1.99)	0.139
SCNN1D	rs609805	G>G/A	0.62	0.31	2.21 (1.92-3.09)	0.001

## DISCUSSION

The present two stage case-controls study screened Pakistani Pashtun population for potential T2DM associated risk variants. In stage first (the discovery phase) Exome Sequencing was performed whereas in the 2nd stage (also called the confirmation/validation stage) the WES reported variants were analyzed for potential T2DM association. WES involves sequencing the protein-coding regions of the genome, which are believed to harbor a significant portion of disease-related variants. Exome sequencing detected novel variants in the following genes namely *ANKRD65, TTLL10, RNF223* and *SCNN1D* and known genetic variants in *NOTCH2, GCKR, NEUROD1, CAPN10, ADCY5, WFS1, CDKAL1, HLA-B, GCK, PAX4* and *SLC30A8* genes and marked/labeled these as pathogenic and non-pathogenic. We focused novel variants compared to known variants. Among the prioritized/novel variants the variant rs1781133 (c.1165C>A, p.Glu389Gln) is present at exon 4 of the *ANKRD65* gene. This alteration results from substitution of glutamic acid at position 389 with glutamine, and has shown significant association with T2DM in the study population. Although the clinical significance of this alteration remains unclear and conflicting. We observed notable association of *ANKRD65* gene with T2DM other studies reported it for neurodevelopment disorders.^13^,^14^ It’s important to note that the clinical significance of a genetic variant can vary across different populations and contexts.

Likewise SNP rs2274791/*TTLL10* (c.1733G>A, p.Gly578Asp) which results from substitution of Glycine at position 578 with aspartate showed positive association with T2DM in the study population. Hu.et.al in Taiwanese cohort reported the same variant (rs2274791/*TTLL10*) for a neurological movement disorder dystonia.^15^ It suggests that this variant may have pleiotropic effects. Pleiotropy refers to a single genetic variant influencing multiple phenotypic traits or diseases. Mutation (rs71628928) in *RNF223* gene also showed to increase risk for T2DM in the study population. The variant rs71628928 (c.725C>A, p.Pro242His), represents genetic change that occurs at position 725 in the DNA sequence. This change leads to the substitution of a cytosine (C) nucleotide with an adenine (A) nucleotide, resulting in a change at the protein level. Specifically, the amino acid proline (Pro) is replaced by histidine (His) at position 242 of the protein. It is suggested that mutations in *RNF223* gene increase risk for pancreatic cancer which forward effect insulin secretion from pancreas and increase chances of getting T2DM.^16^ SNP rs609805 (G>G/A) located on *SCNN1D* gene was marked pathogenic for T2DM in our study population. This genetic variant results in a change at the protein level where the amino acid glycine (Gly) is replaced by arginine (Arg) at position 770. Moreover, *SCNN1D* encodes for the delta subunit of the epithelial sodium channel (ENaC), which plays a role in sodium ion transport across epithelial cell membranes. Variants in *SCNN1D* have been associated with various other pathological conditions, including hypertension and Liddle syndrome.^17^,^18^

Among the known reported variants (Table-III); SNP rs1801262/*NEUROD1* was marked pathogenic by WES for T2DM based on SIFT (Sorting Intolerant from Tolerant) and PolyPhen (Polymorphism Phenotyping) score. The *NEUROD1* gene encodes for the neurogenic differentiation one protein, which is a transcription factor involved in the development and function of pancreatic beta cells. Variants in NEUROD1 have been studied in relation to various conditions, particularly those related to impaired glucose metabolism and diabetes.^19^ Likewise WES detected three variants rs7607759, rs55878652, and rs2975766, are located in the *CAPN10* (Calpain 10) gene as pathogenic for T2DM in the target population. *CAPN10* has been studied extensively in relation to Type-2 diabetes mellitus (T2DM) due to its potential involvement in glucose metabolism and insulin signaling.^20^,^21^ Exome sequencing also reported rs4482616/*ADCY5* deleterious in the study population. *ADCY5* encodes for the enzyme adenylate cyclase 5, which plays a role in the production of cyclic adenosine monophosphate (cAMP), a molecule involved in cellular signalling. A number of studies have reported polymorphism in *ADCY5* for T2DM association.^22^,^23^

Two SNPs namely rs1801212 and rs1801208 located on the WFS1 (Wolfram syndrome 1) gene were detected pathogenic for T2DM in the study population. Polymorphisms in the *WFS1* gene have been associated with a rare genetic disorder called Wolfram syndrome, also known as DIDMOAD (Diabetes Insipidus, Diabetes Mellitus, Optic Atrophy, and Deafness) syndrome.^24^ Variant rs77152992/*CDKAL1* and two variants rs712701, rs772936097 located on *PAX4* were also found pathogenic for T2DM in the target population. *CDKAL1* is involved in the regulation of cell division and insulin secretion. Variants in the *CDKAL1* gene have been associated with an increased risk of developing T2DM.^25^-^27^ *PAX4* is a transcription factor that plays a critical role in pancreatic development and the regulation of beta cell function. Polymorphisms in PAX4 have been identified and investigated in relation to T2DM susceptibility and beta cell dysfunction.^28^

### Study Strength and future suggestions:

The present studied identified novel variants in ANKRD65, TTLL10, RNF223, and SCNN1D, which have not been previously reported with T2DM. This expands the current understanding of the genetic architecture of T2DM and opens new avenues for research. Future large scale genomics studies are suggested to identify the pathway how these newly identified pathogenic variants contribute to T2DM pathogenesis.

### Limitations:

The study included only Pashtun participants from Khyber Pakhtunkhwa province, the inclusion of participants from other Pakistani sub-populations such as Punjabi, Sindhi, and Balochi, would have enhanced the diversity and generalizability of the study findings. Secondly sample size was limited to one thousand participants.

## CONCLUSION

This study confirmed the positive association rs1781133/ANKRD65, rs2274791/TTLL10, rs71628928/RNF223, and rs609805/SCNN1D with T2DM. Looking to high prevalence of T2DM in Pakistani population it is recommended that further studies should be designed with larger sample size to help develop more effective treatment approaches and personalized therapies tailored to individuals’ genetic profiles.

### Author’s Contribution:

**AJ, RAM, JYK, TM, MK, SSA, NR** and **ARA:** Conception, design, acquisition of data, analysis and interpretation of data, methodology, validation, original draft writing and revising. **AJ, TM, MK** and **SSA:** Formal analysis. **AJ, NR** and **ARA:** Investigation. **AJ:** The principal investigator is responsible and accountable for the accuracy or integrity of the work.

All authors have read and approved the final manuscript.
